# Do You Know What You Drink? Comparative Research on the Contents of Radioisotopes and Heavy Metals in Different Types of Tea from Various Parts of the World

**DOI:** 10.3390/foods13050742

**Published:** 2024-02-28

**Authors:** Elżbieta Grządka, Anna Bastrzyk, Jolanta Orzeł, Agata Oszczak-Nowińska, Bartłomiej Fliszkiewicz, Mateusz Siemieniuk, Krzysztof Sobczyński, Olgierd Spławski, Katarzyna Gołębiowska, Oskar Ronda, Bartłomiej Michał Cieślik

**Affiliations:** 1 Institute of Chemical Sciences, Faculty of Chemistry, Maria Curie-Sklodowska University, M. Curie-Skłodowska Sq. 3, 20-031 Lublin, Poland; jolanta.orzel@mail.umcs.pl (J.O.); katarzyna.golebiowska.kg@gmail.com (K.G.); 2 Department of Process Engineering and Technology of Polymer and Carbon Materials, Faculty of Chemistry, Wroclaw University of Science and Technology, C. K. Norwida 4/6 Sq., 50-373 Wroclaw, Poland; anna.bastrzyk@pwr.edu.pl; 3 Institute of Chemistry, Military University of Technology, Kaliskiego 2 Str., 00-908 Warszawa, Poland; agata.oszczak@wat.edu.pl (A.O.-N.); bartlomiej.fliszkiewicz@wat.edu.pl (B.F.); 4 Department of Analytical Chemistry, Faculty of Chemistry, Gdańsk University of Technology, Gabriela Narutowicza 11/12 Str., 80-233 Gdansk, Poland; mati.siemi@gmail.com (M.S.); k.sobczynski912@gmail.com (K.S.); olgierd529@gmail.com (O.S.); oskar.ronda@pg.edu.pl (O.R.); bartlomiej.cieslik@pg.edu.pl (B.M.C.)

**Keywords:** tea, radioisotopes, heavy metals, health risk assessment

## Abstract

The aim of this study was to assess the potential health risks of radioactive elements and heavy metals ingested through the consumption of various types of tea imported to the Polish market (black, green, red, oolong and white). The concentrations [Bq/kg] of radionuclides (^40^K, ^137^Cs, ^226^Ra, ^210^Pb and ^228^Th) in tea leaves before and after brewing were measured using γ-ray spectrometry with high-purity germanium (HPGe). The concentrations [mg/kg] of the studied elements (Fe, Cr, Cu, Mo, Al, Mn, Ni, P, V, Cd and Pb) were determined using a microwave-induced plasma optical emission spectrometer (MIP-OES). The results presented here will help to expand the database of heavy metals and radioactivity in teas. With regard to the potential health risk, the percentage of leaching of individual elements in different types of tea infusions was determined, and the assessment of the consumption risk was estimated. Since the calculated exposure factors, namely the HQ (Hazard Quotient) and THQ (Target Hazard Quotient), do not exceed critical levels, teas can still be considered health-beneficial products (most of the radionuclides as well as elements remain in the leaves (65–80%) after brewing).

## 1. Introduction

Tea is one of the most popular and widely consumed beverages in the world today, with an estimated worldwide consumption of 18–20 billion cups of tea per day [1,2]. This beverage is made from the leaves of the tea plant *Camellia sinensis*, a member of the *Theaceae* family. The tea plant originally comes from Southwest China and Eastern India. Most tea plants grow in tropical and subtropical regions with good drainage and acidic soils where the pH of the soil is suitable for tea plant growth and is between ~4 and 6.5 [3]. Currently, it is also grown in many other places around the world, especially in Asia, Africa, the Middle East and South America. China is the largest tea producer in the world, followed by India, Sri Lanka, Kenya, Turkey, Indonesia and Japan.

Teas are categorized into three main types according to the production process. A distinction is made between non-fermented green tea, semi-fermented oolong tea and fully fermented black tea [4,5]. In addition to these main types, there are others such as white, yellow and pu-erh. The “fermentation” of tea occurs during the manufacturing process and refers to the enzymatic oxidation catalyzed by polyphenol oxidase [6,7]. Each type of tea not only has a unique appearance and flavor, but also a unique chemical composition. The methods of producing tea are also different. To prepare green tea, fresh tea leaves are steamed or fried in a pan. These methods inactivate the tea enzymes and prevent the oxidation of tea polyphenols [8]. Oolong tea is semi-fermented to achieve a medium level of enzymatic oxidation (between 8% and 85%) and then dried. Black tea is produced by crushing the tea leaves to promote the enzymatic oxidation of the tea polyphenols in a fermentation process. White tea is made from the buds and leaves of young plants. These plant parts are wilted in natural sunlight and then lightly processed to prevent further oxidation [1]. During the preparation of yellow tea, the drying phase is slower than that for green tea, and the moist leaves are left to rest until they turn yellow. Pu-erh tea is stored in the air for a period of months to years. Taking into account customers’ preferences for teas, green and black teas are the most popular, accounting for about 21–77% of global tea consumption [1].

Apart from its delicious flavor, tea is also known for its therapeutic properties resulting from its chemical and biochemical compositions [9]. Tea contains many different chemical compounds, such as alkaloids (caffeine, theobromine and theophylline), volatile acids, polyphenols such as catechins and flavonoids, polysaccharides, proteins, amino acids, lipids, vitamins and many others [10,11]. Most tea components have positive effects on human health, for example, strengthening the immune system; having an antioxidant effect; lowering cholesterol levels; having anticarcinogenic, antimicrobial and antidiabetic potential; and reducing the risks of heart disease, stroke, and certain types of cancer [12,13]. However, the actual effect of tea on human health is still controversial, as this beverage may also contain some hazardous components [14,15,16,17], which can also be found in other natural drinkable matrices such as coffee, milk, or wine [18,19,20]. Toxic metals (e.g., Hg, As, Cd, Ni and Pb) as well as radioactive elements and pesticides can be found in dry plant material [21,22]. It should be emphasized that there are also some metals that are useful for human health (Cu and Zn). Their concentrations differ depending on many factors but should not be exceeded. The tea plant is able to intake some chemical substances, such as heavy metals, fluorine and radioisotopes from the soil, which accumulate in its leaves [16,23,24]. Therefore, considering the high consumption of tea around the world, the levels of hazardous compounds in teas should be carefully analyzed. It should be mentioned that the contamination of tea leaves mainly occurs due to the chemical compositions and physicochemical properties of the soils in which plants grow, and the plant’s metabolism as well [25,26,27]. Obtaining knowledge on the absorption and accumulation levels of heavy metals in tea leaves and, thus, in tea infusion is crucial because the infusion rate of particular heavy metals varies between 10% and 60%, with about 90% of infused heavy metals entering the gastrointestinal tract [28,29,30]. The intake of toxic elements by the human body depends on the total element contents in the tea leaves, the proportion of element contents extracted to the infusion and the bioavailability of the particular element [2,9]. Lead (Pb), mercury (Hg) and cadmium (Cd) are the most important toxic metal pollutants that can be detected in plants [21]. They reveal negative effects on the immune, nervous and reproductive systems [12]. Regarding radionuclides, there are two sources of radionuclides in the environment: (i) natural (mainly from the ^238^U and ^232^Th series and natural ^40^K) and (ii) artificial ones (as ^137^Cs long-lived product of nuclear weapon testing). Radionuclides can enter the human body via the food chain. The soil–plant–human sequence is an important route of radionuclide transfer into the human body. In accordance with international legal arrangements, it is necessary to assess the radiation doses received in the population of each country [31,32]. Furthermore, tea plants can be treated as sensitive indicators of environmental contamination with radionuclides, as they have a tendency to absorb minerals also containing radionuclides [33]. It has been estimated that the effective dose from tea consumption is relatively low compared to the United Nations Scientific Committee on the Effects of Atomic Radiation (UNSCEAR) reference limit which has an ingestion dose amounting to 290 μSv/year [31]. Considering that daily tea consumption is around 4 g per person, there is no radiological risk caused by tea consumption. However, this contribution could not be entirely negligible in the case when other dietary ingredients are contaminated with radionuclides.

Drinking tea is very popular in Poland. According to statistical data, the average Polish citizen drinks 94 L of tea per year [30,34], which puts them in third place in Europe in terms of tea consumption after British and Irish people who drink 201 and 173 L a year, respectively. Therefore, the main purpose of this study was to analyze the elemental compositions of the tea samples available on the Polish market. The largest groups analyzed were the most popular black and green teas, but niche varieties such as white, red and oolong teas were also analyzed. The detailed objectives of the studies presented were (i) to determine the concentrations of toxic metals (Cr, Cd and Pb) and other elements (Al, Cu, Fe, Mn, Mo, Ni, V and P) in commercial tea samples of different varieties; (ii) to estimate the concentrations of radioisotopes in commercial tea samples of different varieties; (iii) to evaluate the potential risks to human health from these microelements and radioisotopes in tea infusions; and (iv) to establish a relationship between the tea variety and its origin and its potential risks to human health.

## 2. Materials and Methods

### 2.1. Samples Preparation

The tea samples from various types of tea were imported to the Polish market from 2021 to 2023. Tea samples were tested before and after brewing without any pre-treatment (Table 1). The infusion procedure was as follows: A 20 g sample was poured into 1 L of redistilled water at a specified temperature (black tea at 100 °C, pu-erh and oolong teas at 85–90 °C and green tea at 75 °C). The European Union legislation stipulates that 20 g of tea should be brewed with 1 L of water (resulting in a dilution factor of 50). Redistilled water was used to avoid the influence of contaminants from tap water. Black, green, red and oolong teas were brewed for 3 min, but white tea was brewed for 5 min. After brewing, the infusion was separated by decanting and finally filtering. Then, the wet teas were dried in a laboratory dryer, first at 70 °C (24 h) and then at 50 °C until completely dry (about 24 h).

### 2.2. Radionuclide Determination in Tea Samples

Before the measurement, the samples were stored at room temperature in tightly sealed containers for 30 days to allow an equilibrium to develop between radon and its progeny. The measurements were carried out in a disc geometry. The supplied material was weighed with an accuracy of 0.0001 g and placed in a plastic cup with a diameter of 60 mm and a height of 15 mm. The measurement time was 24 h for each sample. All gamma spectrometry measurements were performed with a Canberra spectrometer equipped with a semiconductor detector HPGe (Canberra GC3018), a relative efficiency of 30%, an FWHM resolution of 1.80 keV (for the 1.33 MeV line), an energy range of 40 keV to 2.2 MeV and a peak/Compton ratio of 58:1. The detector was electronically cooled (Canberra Cryo-Pulse 5 Plus), which kept the detector at an operating temperature of about −185.0 °C. Canberra’s Gennie 2000 (gamma analysis software) was used to acquire and analyze the data, with the measurement uncertainty defined as 1 sigma (one standard deviation).

### 2.3. Elemental Analysis of Tea Samples

#### 2.3.1. Sam

Prior to elemental analysis, mineralization of the samples was carried out as follows: First, 1 g of each tea sample was weighed and placed in Teflon vials containing 8 mL nitric acid (67%, highest purity, provided by Merz, Frankfurt am Main, Germany). The Multiwave GO microwave digestion system from Anton-Paar was used. In the first step of mineralization, heating at 125 °C for 20 min was carried out to avoid a drastic increase in temperature. In the next steps, a proper mineralization lasting 30 min at 185 °C was carried out. Details of mineralization procedure are presented in Table 2 in the Supplementary Materials. The relative pressure associated with the durability of the safe valves was not exceeded (Table 2). The solutions obtained were filled to 10 mL and made up with demineralized water and then analyzed.

#### 2.3.2. Microelement Determination

A microwave-induced plasma optical emission spectrometer (MIP-OES), namely model MP-AES 4210 from Agilent, was used to determine the concentrations of the microelements. The following operating parameters were used: a pump speed of 15 rpm, a sample acquisition time of 100 s and a plasma stabilization time of 50 s, a purge time between samples of 45 s (1–3% HNO_3_ was used as rinsing liquid). Reference solutions from Sigma-Aldrich (St. Louis, MO, USA) were used to prepare the calibration solutions. Automatic background correction was performed for all 4 repetitions used to determine the elements at each wavelength. Wavelengths and detection limits are listed in Table 3.

#### 2.3.3. Consumption Risk Assessment Calculations

The consumption risk assessment was only calculated for microelements, as the concentrations of the most important radionuclides (^238^U and ^232^Th) were very low. Based on the analysis of the results obtained, a health risk assessment was prepared for selected elements based on the guidelines proposed by the US Environmental Protection Agency (US EPA). This method takes into account three types of intake: skin contact, oral intake and inhalation. For tea, the risk was assessed on the basis of oral intake. First, the average daily intake (ADI) was calculated using the following formula [35]:

(1) $$ADI=\frac{C\times IR\times EF\times ED}{BW\times AT}$$

where C—the content of a given element in tea leaves [mg/kg]; IR—the index of tea consumption (assumed value 11.4 g/day) [kg/day] [36]; EF—the frequency of exposure (assumed value 365 days/year) [day/year]; ED—the duration of exposure in life years (assumed value 57 years); BW—the average human body weight in kilograms (assumed value 61.75 kg) and AT—the annual exposure period calculated as ED·365 days.

The hazard quotient (HQ) and the total hazard quotient (THQ) were then calculated [35].

(2) $$HQ_{i}=\frac{ADI}{RfDi}$$

(3) $$THQ=\overset{n}{\underset{i=1}{\sum }}HQ_{i}$$

where RfDi—the maximum acceptable dose of a toxic substance taken orally of the i-th element [mg/kg·day].

## 3. Results and Discussion

**Figure 1:**
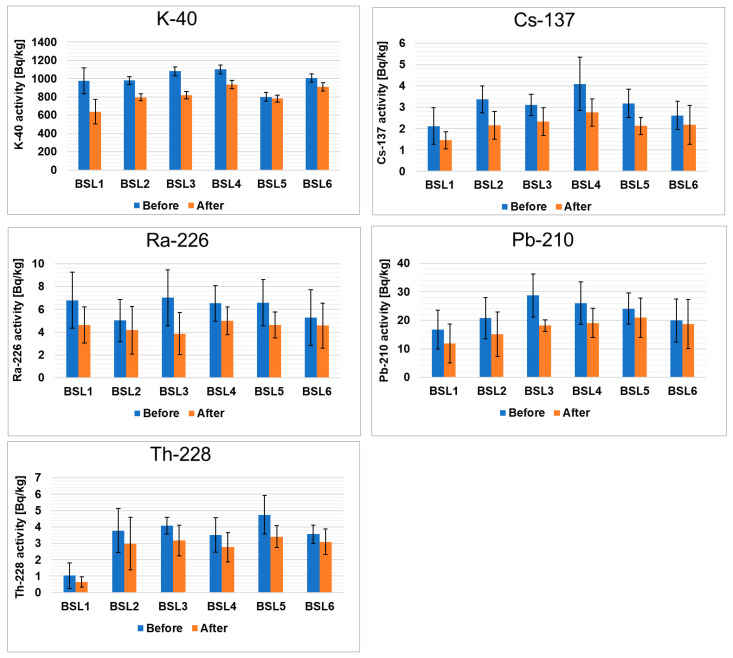
Activities of radioactive elements in black teas from Sri Lanka before and after the brewing process.

Figure 1 presents the activity of radioactive elements in black teas from Sri Lanka before and after the brewing process. The concentrations of the radionuclides were given in Bq/kg dry weight. The results of examining the radionuclide contents in black teas are in the following order: ^40^K > ^210^Pb > ^226^Ra > ^228^Th > ^137^Cs. In the tea samples, the activity of ^40^K ranged from 976 ± 134 Bq/kg to 1100 ± 48 Bq/kg before brewing and from 638 ± 135 Bq/kg to 934 ± 45 Bq/kg after brewing. It should be emphasized that all gamma radioactivity in the samples of herbaceous plants originates from natural isotopes, mainly ^40^K [37]. The potassium content in teas is the highest compared to other radioelements, which is due to the fact that this element is the seventh most abundant element in the earth’s crust and accounts for 2.6% of the weight of the earth’s crust [38]. ^40^K is responsible for about 60% of the total annual effective dose [39]. It is known that natural potassium contains about 0.0117% ^40^K, and plants absorb about 88–96% of potassium from the soil through the root system [40,41]. Therefore, the significant concentration of ^40^K in tea leaves is the result of specific metabolic processes during plant growth and the application of potassium fertilizers [42,43]. The comparison between all measured samples shows that the lowest amount of this nuclide was detected in the BSL5 sample and the highest was detected in the BSL4 sample. The degree of release of ^40^K during the brewing of these teas ranges from 1 to 38%. The tea samples analyzed do not pose a risk to public health as these radioactivity levels are well below the recommended safety limits [31]. There are several factors that can influence the extraction process of teas. The most important are the mass ratio of tea to water used for brewing and the ionic charge of the radionuclides [44]. In our case, all teas were brewed with the same mass ratio, namely 1:50.

The activity of ^210^Pb ranged from 17 ± 7 Bq/kg to 29 ± 7 Bq/kg before brewing and from 12 ± 7 Bq/kg to 21 ± 7 Bq/kg after brewing. The Pb-210 isotope is a natural one and represents the ^238^U decay series, but it can also be released into the environment through human activities [45]. The reason for the presence of ^210^Pb in the tea leaves is its uptake from the soil by the root system and the direct deposition of contaminated dust on the leaves of the plant [46,47]. The presence of radon gas in atmospheric air may also be an additional source of ^210^Pb, also called “unsupported”. Lead can accumulate in the humus-rich surface layers of the soil, as it is easily incorporated into organic matter [48]. Considering that the tea leaves are not washed before processing, these processes may contribute to an increase in the ^210^Pb content in tea. Furthermore, the ^210^Pb concentration in tea leaves is related to the degree of industrialization of the area where the tea is grown [45,49]. The lowest level of this nuclide was reported for the BSL1 sample and the highest was reported for the BSL3 sample. In the case of ^210^Pb, the level of release ranges from 10 to 39%.

^137^Cs, an anthropogenic nuclide that appeared after the nuclear weapon tests and Chernobyl disaster, was detected in traces. The activity of ^137^Cs ranged from 2.1 ± 0.9 Bq/kg to 4.1 ± 1.3 Bq/kg before brewing and from 1.5 ± 0.4 Bq/kg to 2.8 ± 0.6 Bq/kg after brewing. In the case of ^137^Cs, the international radionuclide action level for food is 100 Bq/kg [31]. The uptake of ^137^Cs in tea leaves is the result of the absorption of this isotope from the soil. Differences in the ^137^Cs levels in plants are due to several factors. The most important ones are the level of soil contamination; physicochemical properties of soil (i.e., humic substance presence, a share of mineral components containing exchangeable sites for Cs^+^); meteorological conditions; biological activities of microbes and small soil animals (as earthworms), so-called “bioturbation”; fertilization level and type of fertilizer [47]. The ^137^Cs is present in the soil solution in the form of cation, which means that, under favorable pH conditions, a cation exchange mechanism is responsible for facilitating isotope migration from the soil to plant [50]. Plants take up cesium relatively easily, and in the solution, it is present as a monovalent cation. Tea plants usually have large leaves and a relatively well-developed root system. Therefore, a plant’s ability to absorb substances from the soil is considerable. It was established that the lowest concentration of this nuclide was seen in the BSL1 sample and the highest was seen in the BSL4 sample. During brewing, only 15–32% of ^137^Cs is released into the tea infusion, while 68–85% of this nuclide remains in the tea leaves.

Another nuclide detected in the leaves of black tea produced in Sri Lanka is ^228^Th. It is usually a product of the decay of ^228^Ra, which is absorbed together with ^226^Ra. The lowest content of this nuclide was found in sample BSL1 and the highest was found in sample BSL5. The amount of this nuclide in the analyzed samples was between 4.7 ± 1.2 Bq/kg and 1.0 ± 0.8 Bq/kg, with 14–40% being released during a brewing procedure. 

Radium is ubiquitous in the environment and can be found in water, rock, soil and vegetation. ^226^Ra is a member of the uranium-238 decay series and is an alpha emitter with a half-life of 1600 ± 7 years [51]. The typical world value for the activity concentration of ^226^Ra in soil is given as 35 Bq/kg [52]. In uncontaminated soils, the uptake of radium by a plant depends on the presence of other alkaline earth elements with small ionic radii, such as barium, strontium, calcium and magnesium. The higher the concentrations of these elements in soil, the lower the uptake of radium by plants [51]. Figure 1 shows that the maximum ^226^Ra activity was detected in BSL3 and did not exceed 7.0 Bq/kg, while the lowest activity value of this nuclide was 5.0 Bq/kg in BSL2. After brewing, the maximum and minimum ^226^Ra activities in tea leaves were 5.0 ± 1.2 and 3.9 ± 1.9 Bq/kg, respectively, corresponding to a release level of 10–43% into the infusion.

Comparing the radioisotope content of black teas from Sri Lanka (Figure 1) with black teas from India (Figure S1) and other parts of the world (Figure S2), we found that these values are quite similar. However, it is noticeable that the 40K content is lower in the teas from Africa (BK1) and mixed teas (BM1, BM2 and BM3); the other differences are not statistically significant.

**Figure 2:**
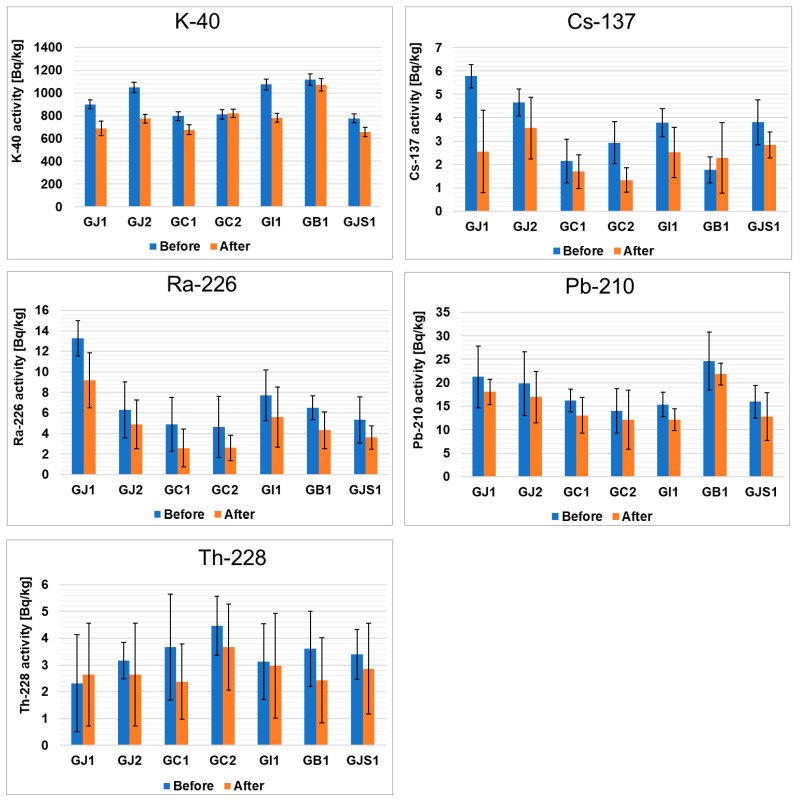
Activities of radioactive elements in green teas from different parts of the world before and after the brewing process.

Interesting conclusions can be drawn from the comparison of the isotopic compositions of green and black teas (Figure 1 and Figure 2). In green teas, the concentrations of ^137^Cs and ^226^Ra are higher, while the concentration of ^210^Pb is lower. In the case of ^228^Th, no statistically significant changes were observed. As in the case of ^40^K, the percentage of its release into solution after the brewing process was the highest in the GB1 sample (about 27%), while the lowest value was observed in the GC2 sample (values below the detection limit). It should be emphasized that although the concentration of ^40^K is lower in green teas than in black teas, more of this isotope is released into the infusion. In the case of ^137^Cs, a much higher percentage of this radioisotope is also released in green teas than in black teas. Most of the ^137^Cs was washed out during the brewing of the GJ1 sample (56%), while the percentage was almost negligible for the GI1 sample. This observation is consistent with the data in the literature [44], according to which the leaching of ^137^Cs during tea brewing is usually between 50 and 70%. In contrast to the radioisotopes mentioned above, ^226^Ra and ^210^Pb are less leached from green teas. For ^226^Ra, the highest leaching percentage was found in the GJ1 sample (31%), and the lowest was found in the GJ2 sample (22%). Interestingly, the leaching percentage of ^210^Pb was similar in all samples and was around 20%. Considering the ^228^Th isotope leaching results showed that most of this element is leached in GC1 tea (35%), but there are also samples where leaching is close to zero (GJ1), and these values are comparable to those obtained for black teas.

Less popular teas, such as red, oolong and white teas, were also analyzed (Figures S3–S5). According to the data obtained, the highest concentrations of ^40^K occurred in white teas, while the concentrations of this radioisotope in oolong and red teas were similar to those in the previously analyzed black and green teas. As far as ^137^Cs and ^228^Th are concerned, the concentrations of these nuclides were very similar in all of the teas analyzed. In the case of ^226^Ra, the concentrations in red teas were similar to those in green teas, but higher than the results obtained for black teas. The ^210^Pb content in red teas was quite high, but comparable to black and white teas. In terms of the release levels, the concentrations of ^210^Pb in oolong tea and of ^228^Th and ^137^Cs in red tea were similar to those in black tea. However, for ^40^K and ^226^Ra (red, oolong and white tea), ^210^Pb (red and white tea) and ^228^Th (oolong and white tea), lower values were found than for black tea. Nevertheless, ^210^Pb was leached more effectively from green tea than from oolong tea. Red tea, on the other hand, released less ^228^Th, ^40^K, ^137^Cs and ^210^Pb in the infusion compared to green tea. In the white tea samples, the extent of ^210^Pb release was comparable to that of green tea. The values determined for ^228^Th, ^137^Cs, ^226^Ra and ^228^Th were lower than in those the green tea samples.

**Figure 3:**
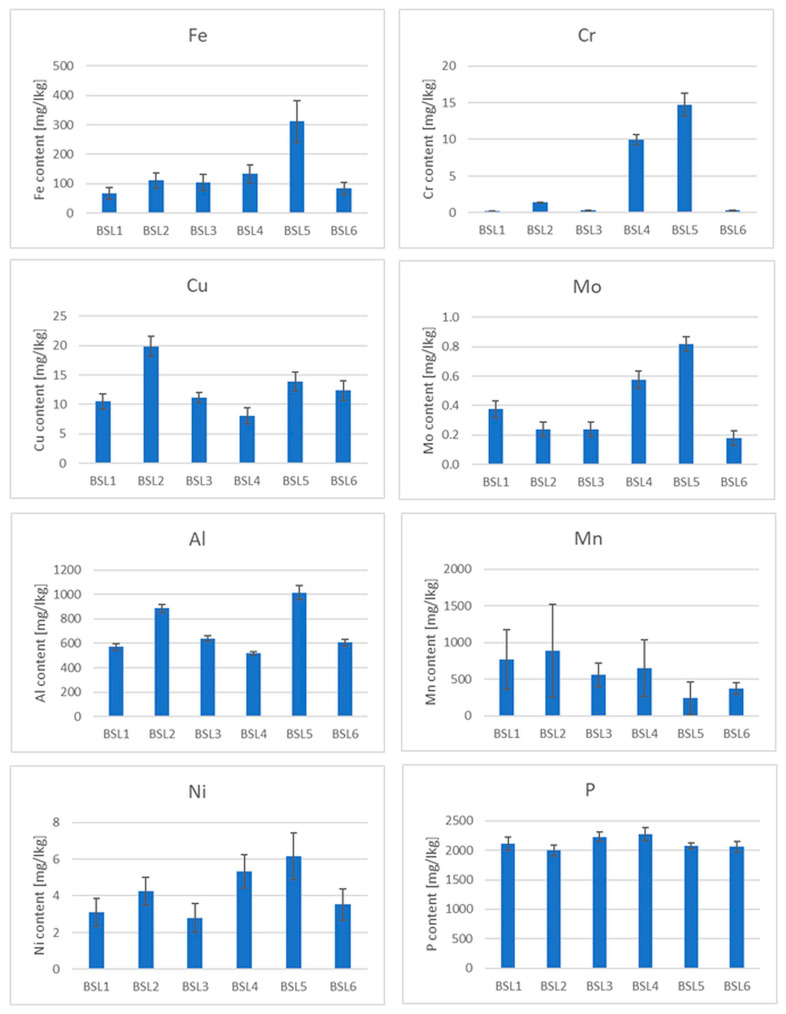
Element contents in black teas from Sri Lanka.

**Figure 4:**
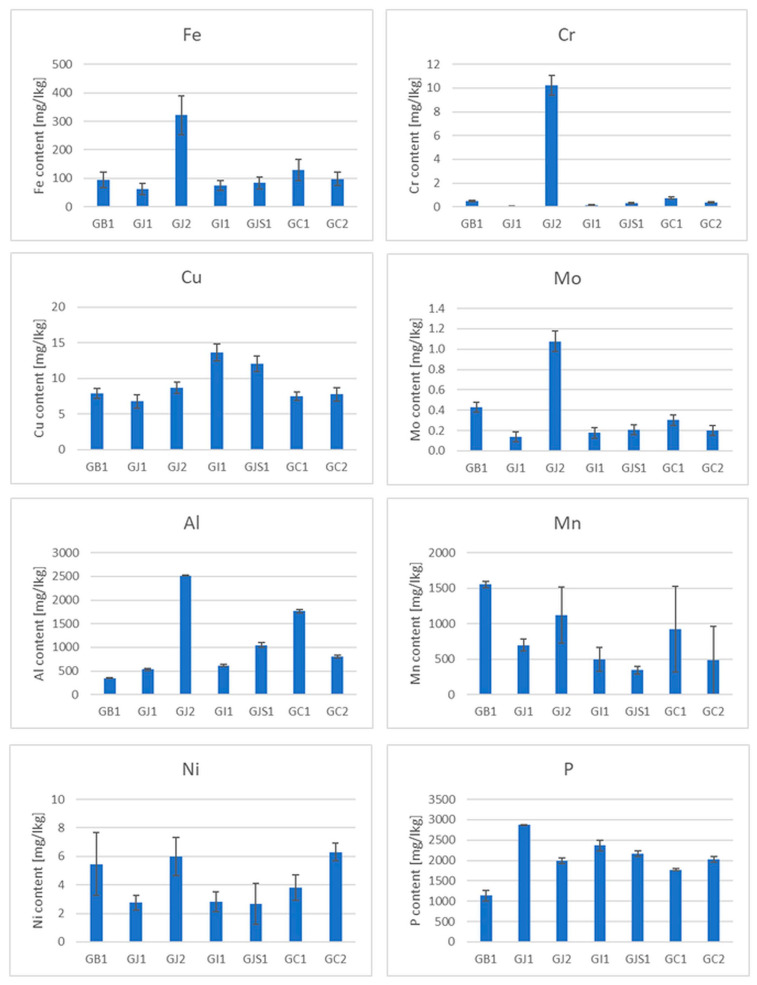
Element contents in green teas from different parts of the world.

The presence of some micro and macro elements (Fe, Cr, Cu, Mo, Al, Mn, Ni, P, V, Cd and Pb) in different types of tea was also estimated in the samples (Figure 3, Figure 4 and Figures S6–S10). Special attention was paid to heavy metals, whose presence in the body can pose the greatest danger (Pb, Cd and Cr), and Al, which is highly abundant in soils and reveals a rather toxic effect on human and plant bodies. Three elements whose concentrations were analyzed revealed values below the limit of detection (LOD) in the case of all samples. The values of LOD were established as 0.24 mg/kg for lead, 0.051 mg/kg for cadmium and 0.02 mg/kg for vanadium. Therefore, these elements were not shown and not taken for further consideration. It should be mentioned that tea, which is known to contain antioxidants (e.g., polyphenols), also contains pro-oxidant compounds that are capable of causing oxidative reactions, leading to the unfolding of proteins and DNA damage, e.g., iron ions, copper ions and heavy metals [53], which is very important in the context of the presented research.

**Figure 5:**
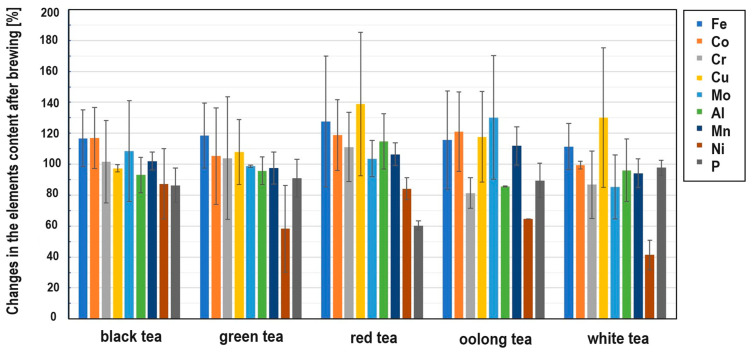
Changes in the element contents after brewing different types of tea.

Iron is an important microelement which is responsible for a number of metabolic processes in the human body. Iron enters the active centers of enzymes and is also a component of many proteins. The main task of iron is oxygen transport, which is made possible by the presence of this trace element in hemoglobin and myoglobin. It has long been known that tea has a negative effect on the absorption of non-haem iron in the diet due to its polyphenol content [54]. The iron content of the black teas from Sri Lanka was about 100 mg/kg. Only in the BSL5 sample was this value the highest among the black teas (Figure 3), but still within the range. Among the green teas, the highest content was found in the GJ2 sample. Surprisingly, the iron content in some of the samples tested was below 100 mg/kg, which could be due not only to the tea variety but also to the geological location of the plant [55]. The analysis of the data in Figure 5 shows that iron is practically not leached out during the brewing process. The reason for this is the presence of flavonoids in the tea infusion [56].

As far as the chromium content in teas is concerned, due to the different and opposing influences of Cr(III) and Cr(VI) on the human body, this element can demonstrate both positive and unfavorable effects on human health. Chromium(III) is responsible for glucose metabolism, it controls blood cholesterol levels, stimulates protein synthesis and suppresses hunger [57]. On the other hand, Cr(VI) is toxic and carcinogenic to humans [58]. In addition, this microelement can be absorbed by plants from the contaminated soil or irrigation water [59]. The Cr content in black tea is much higher than that in other types of tea [60]. The total Cr content in black tea from Sri Lanka (Figure 3) varies between a few and 14 mg/kg, which is consistent with the literature [61]. The highest Cr content is observed in the BSL5 and BI1 samples. With regard to the leaching of Cr during the brewing process, it should be noted that this element is practically not leached from black, green and red teas, but is leached from oolong and white teas, although the Cr content in the latter two types of teas is relatively low.

Copper is also an essential trace element because it acts as a cofactor in enzymes [62]. However, in high concentrations, it can be dangerous and cause stomach disorders, hypoglycemia and dyslexia [63,64]. It should be emphasized that Cu contamination may originate from Cu-based fungicides, which are frequently used in tea gardens [65]. The copper content in the measured samples generally varies from a few to a dozen mg/kg, which is consistent with the data in the literature [55]. However, the content is the highest in the black tea sample BIr1 (about 24 mg/kg). The reason for this could be the high Cu contamination of Iranian soil. Also, in this case, it can be observed that Cu is practically not leached from black, green and red teas, but partially from oolong and white teas.

Molybdenum (Mo) is an essential element that occurs naturally in foods (legumins, papaya and whole grain) and is available in food supplements. It is a component of four enzymes that break down proteins and alcohol [66]. Although molybdenum can be toxic to animals, it appears to be non-toxic to humans [67]. Studies on the Mo content in tea before and after brewing are very limited. The measured content of this element in the analyzed teas is less than 1 mg/kg. It can also be said that Mo is practically not leached out of the tea during brewing. Minimal leaching was only observed in white teas.

Tea is one of the few plants capable of accumulating aluminum (Al) [68]. According to the latest findings, tea plants require this element for root growth and the development of tea leaves, which are known to be hyperaccumulators of aluminum [69]. It is possible that Al is used by plants to improve the uptake and utilization of phosphorus [70]. Although it is a useful component for plants, it may pose a danger to humans (as a neurotoxicant) [68]. The average total daily intake of Al in Europe is a few milligrams per day [68]. Considering that the typical Al content in tea infusions is 1–6 mg L^−1^, tea is an important source of dietary Al intake [68]. In the black tea samples tested, the aluminum content was between 200 and 1500 mg/kg. According to the literature, the aluminum content varies between 120 and 3300 mg/kg [71]. On the other hand, the aluminum content in green teas was higher and ranged from 400 to 2500 mg/kg, the content in red teas about 1000 mg/kg and the content in white teas was 200–600 mg/kg. Similar to the previously discussed elements, aluminum is readily leached from oolong tea, but is practically not leached from black, green, red and white teas.

When it comes to manganese (Mn) in the diet, tea is also one of the most important sources for the absorption of this element by the human body. Manganese is a microelement that plays an important role in the proper functioning of photosynthesis in plants by activating enzymes that are important for the synthesis of chlorophyll. It is also involved in the regulation of a plant’s hormonal balance, forms chloroplasts and increases resistance to disease [72]. However, Mn can also be toxic to the body, causing hallucinations, nerve damage, lung embolism and bronchitis [60]. The content of this chemical element in black teas ranges from 200 to 1000 mg/kg, the content in green teas is between 300 and 1500 mg/kg, the content in red teas is between 600 and 1000 mg/kg and the content in white teas is between 600 and 1200 mg/kg. This microelement is only easily leached from white teas.

Nickel (Ni) is a ubiquitous element in the environment. It can be found in both plant and animal foods [73]. In the human body, Ni participates in the decomposition of urea. The average recommended daily intake of Ni through food is about 0.1 to 0.3 mg per day [74]. Although this element is not essential for humans, as a trace element, its effects on health have been studied and it is known that its high concentrations can be toxic to humans [75]. The Ni content in black teas ranges from 3 to 13 mg/kg, with the highest Ni content being found in BIr1 tea. In green teas, the Ni content is 3–6 mg/kg, the content in red teas 5–6 mg/kg, the content in oolong teas 2.5–3.5 mg/kg and the content in white teas 5 to 6 mg/kg. As far as leaching is concerned, this metal leaches quite well from all types of tea, with the most effective leaching being around 60% in white tea, 40% in green tea, 20% in red tea and 10% in black tea.

Phosphorus (P) plays an important role in the development of plants: it is involved in the formation and growth of the root, which, in turn, is the most important element for the proper functioning of a plant and enables its proper development. Due to the insufficient supply of phosphorus from soil, tea growers use P fertilizers every year to increase the productivity of tea [76,77]. The P content in black teas ranges from 1000 to 2800 mg/kg, with the highest P content being observed in BK1 tea; in green teas, the P content is 1100–2900 mg/kg, the content in red teas 2000–2900 mg/kg, the content in oolong teas 1500–2900 mg/kg and the content in white teas 2500–4000 mg/kg. Phosphorus is leached quite well from red tea at about 40%, and from black, green and red teas, the percentage of leaching is about 10%, while the least phosphorus is leached from white tea (a few percent).

### Consumption Risk Assessment

Table 4 shows the THQ and HQ indices calculated only for the elements whose toxicity has been demonstrated in the literature. For the measured elements whose concentrations were <LOD, the influence on the calculated indexes was negligible; thus, they were omitted. As one can see, none of the samples exceeded the THQ threshold of “1”, which would indicate a risk of dangerous health complications. Therefore, based on the THQ and HQi coefficient results presented in Table 4, it can be concluded that the consumption of the tested tea is not expected to have any adverse health effects, except for carcinogenic properties. The authors identified that the uncertainty of determining the THQ coefficient depends, to the greatest extent, on the uncertainty of Mn determination in the sample. As a rule, the uncertainty in determining the THQ coefficient is negligible, but in some cases, such as Mn, which has the greatest impact on the THQ, it may be up to 0.063. Over 90% of all samples have a THQ index of less than 0.6. The highest THQ value (0.6329) was found for the Brazilian tea GB1, while other high values were found for Iran BIr1 and Japan GJ2 teas. The highest average THQ value was found for white (0.4599) and green (0.4327) teas. The value of lead concentration was included as the LOD value in the calculations for almost all samples; therefore, the actual Pb concentration and the target exposure factor are actually lower than the values calculated in this work. However, it is worth noting that the concentrations of Al, Cd, Fe and V were not assessed for the THQ because its concentrations are negligible, the detection limit is extremely low (and does not statistically significantly affect the THQ value) or there are no data on possible hazardous effects. It is also worth noting that, in addition to Cr, Cu, Mn, Mo, Ni and Pb, other potentially toxic elements may be present that can affect the THQ value in the tested tea. Further studies are needed to determine the actual risk of consumption of the tested tea.

## 4. Conclusions

All tested samples of teas contained heavy metal concentrations below the limits specified in the regulations issued in their countries of origin. Otherwise, they could not be commercial products intended for consumption. Still, it was found that the individual elemental impurities determined varied considerably depending on the country of origin and the type of tea. The differences observed could be due to the different levels of contamination in the cultivation areas in the respective countries or due to the potential enrichment properties of certain tea varieties. For a more precise differentiation, a more comprehensive analysis of tea varieties from the same region must be carried out. The analyzed tea samples do not pose a risk to public health as their radioactivity levels are well below the recommended safety limits. However, the UNSCEAR reference dose limit is derived from the total (cumulative) dietary radiation exposure. Therefore, the share of radioactive dose from tea in the cumulative dietary radiation exposure is not completely negligible. The results of the radionuclide contents in black teas are in the following order: ^40^K > ^210^Pb > ^226^Ra > ^228^Th > ^137^Cs. Among the analyzed elements, phosphorus had the highest content in teas, which is not considered a threat due to the significantly lower toxicity of the mentioned non-metallic element compared to the other identified metals. On the other hand, Mo and chromium were found to be the elements with the lowest concentrations among the identified elements. It should therefore be emphasized that some elements were below or close to the detection limit. The calculated exposure factors HQ and THQ do not exceed the critical levels. Thus, teas might still be considered health-beneficial products.

## Figures and Tables

**Table 1:** Descriptions of analyzed tea samples with the places of origin.

	Name	Place of Origin	Acronym
1	Black tea 1	Sri Lanka	BSL1
2	Black tea 2	Sri Lanka	BSL2
3	Black tea 3	Sri Lanka	BSL3
4	Black tea 4	Sri Lanka	BSL4
5	Black tea 5	Sri Lanka	BSL5
6	Black tea 6	Sri Lanka	BSL6
7	Black tea 7	India	BI1
8	Black tea 8	India	BI2
9	Black tea 9	India	BI3
10	Black tea 10	India	BI4
11	Black tea 11	Kenya	BK1
12	Black tea 12	Iran	BIr1
13	Black tea 13	African and Indian teas	BM1
14	Black tea 14	Unknown	BX1
15	Black tea 15	Unknown	BX2
16	Black tea 16	Unknown	BX3
17	Green tea 1	Japan	GJ1
18	Green tea 2	Japan	GJ2
19	Green tea 3	China	GC1
20	Green tea 4	China	GC2
21	Green tea 5	India	GI1
22	Green tea 6	Brazil	GB1
23	Green tea 7	Java and Sumatra	GJS1
24	Red tea 1	China	RC1
25	Red tea 2	China	RC2
26	Oolong tea 1	China	OC1
27	Oolong tea 2	China	OC2
28	White tea 1	China (Fujian)	WC1
29	White tea 2	China	WC2
30	White tea 3	China	WC3

**Table 2:** Mineralization procedure.

Step	Time	Temperature [°C]	Relative Pressure [%]
First step heating	10	25–125	20–60
Stabilization	10	125	60
Second step heating	10	125–180	60–95
Mineralization	20	180	95
Cooling	10	180–60	95–25

**Table 3:** Parameters of MIP-OES analysis.

Element	Wavelength [nm]	LOD [mg/kg]
Al	394.401	1.500
Cd	228.802	0.051
Co	345.351	0.100
Cr	425.433	0.016
Cu	327.395	0.025
Fe	259.940	2.837
Mo	379.825	0.022
Mn	279.827	0.600
Ni	305.082	1.100
P	213.618	11.000
Pb	283.305	0.240
V	309.311	0.020

**Table 4:** Presentation of THQ and HQ for Cr, Cu, Mn, Mo, Ni and Pb.

Tea Label	HQ_Cr_	HQ_Cu_	HQ_Mo_	HQ_Pb_	HQ_Mn_	HQ_Ni_	THQ
BI1	2.03 × 10^−3^	4.95 × 10^−2^	2.71 × 10^−2^	7.68 × 10^−2^	4.09 × 10^−1^	4.09 × 10^−4^	0.5648
BI2	1.32 × 10^−4^	7.30 × 10^−2^	6.09 × 10^−3^	7.68 × 10^−2^	1.98 × 10^−1^	1.71 × 10^−4^	0.3542
BI3	3.35 × 10^−5^	2.44 × 10^−2^	8.86 × 10^−3^	7.68 × 10^−2^	1.09 × 10^−1^	1.10 × 10^−4^	0.2192
BI4	7.85 × 10^−5^	7.35 × 10^−2^	4.66 × 10^−2^	7.68 × 10^−2^	1.97 × 10^−1^	3.11 × 10^−4^	0.3943
BIr1	1.20 × 10^−4^	1.09 × 10^−1^	1.59 × 10^−2^	7.68 × 10^−2^	4.25 × 10^−1^	5.08 × 10^−4^	0.6273
BK1	1.24 × 10^−4^	4.25 × 10^−2^	8.12 × 10^−3^	7.68 × 10^−2^	2.81 × 10^−1^	1.58 × 10^−4^	0.4087
BM1	1.18 × 10^−3^	4.81 × 10^−2^	2.03 × 10^−2^	7.68 × 10^−2^	3.00 × 10^−1^	2.79 × 10^−4^	0.4467
BSL1	2.55 × 10^−5^	4.86 × 10^−2^	1.38 × 10^−2^	8.57 × 10^−2^	1.21 × 10^−1^	1.18 × 10^−4^	0.2692
BSL2	1.71 × 10^−4^	9.17 × 10^−2^	8.86 × 10^−3^	7.68 × 10^−2^	8.82 × 10^−2^	1.61 × 10^−4^	0.2659
BSL3	3.72 × 10^−5^	5.16 × 10^−2^	8.86 × 10^−3^	7.68 × 10^−2^	1.23 × 10^−1^	1.06 × 10^−4^	0.2604
BSL4	1.23 × 10^−3^	3.72 × 10^−2^	2.12 × 10^−2^	7.68 × 10^−2^	8.79 × 10^−2^	2.02 × 10^−4^	0.2245
BSL5	1.81 × 10^−3^	6.41 × 10^−2^	3.02 × 10^−2^	7.68 × 10^−2^	1.39 × 10^−1^	2.34 × 10^−4^	0.3121
BSL6	3.94 × 10^−5^	5.70 × 10^−2^	6.65 × 10^−3^	7.68 × 10^−2^	1.36 × 10^−1^	1.34 × 10^−4^	0.2766
BX1	1.11 × 10^−3^	5.03 × 10^−2^	2.31 × 10^−2^	7.68 × 10^−2^	3.09 × 10^−1^	2.84 × 10^−4^	0.4606
BX2	4.45 × 10^−4^	6.16 × 10^−2^	1.44 × 10^−2^	7.68 × 10^−2^	3.35 × 10^−1^	2.42 × 10^−4^	0.4885
BX3	1.98 × 10^−4^	4.48 × 10^−2^	1.55 × 10^−2^	7.68 × 10^−2^	4.44 × 10^−1^	2.09 × 10^−4^	0.5815
GB1	5.91 × 10^−5^	3.64 × 10^−2^	1.58 × 10^−2^	7.68 × 10^−2^	4.84 × 10^−1^	2.08 × 10^−4^	0.6133
GC1	9.14 × 10^−5^	3.46 × 10^−2^	1.12 × 10^−2^	7.68 × 10^−2^	4.54 × 10^−1^	1.44 × 10^−4^	0.5768
GC2	4.46 × 10^−5^	3.59 × 10^−2^	7.48 × 10^−3^	7.68 × 10^−2^	2.86 × 10^−1^	2.39 × 10^−4^	0.4065
GI1	1.57 × 10^−5^	6.30 × 10^−2^	6.55 × 10^−3^	7.68 × 10^−2^	1.04 × 10^−1^	1.07 × 10^−4^	0.2505
GJ1	4.00 × 10^−6^	3.12 × 10^−2^	5.17 × 10^−3^	7.68 × 10^−2^	1.89 × 10^−1^	1.04 × 10^−4^	0.3023
GJ2	1.26 × 10^−3^	4.02 × 10^−2^	3.97 × 10^−2^	7.68 × 10^−2^	4.55 × 10^−1^	2.28 × 10^−4^	0.6132
GJS1	3.88 × 10^−5^	5.56 × 10^−2^	7.66 × 10^−3^	7.68 × 10^−2^	8.96 × 10^−2^	1.01 × 10^−4^	0.2298
OC1	5.42 × 10^−5^	3.66 × 10^−2^	9.78 × 10^−3^	7.68 × 10^−2^	2.29 × 10^−1^	1.27 × 10^−4^	0.3524
OC2	6.31 × 10^−5^	3.52 × 10^−2^	7.66 × 10^−3^	7.68 × 10^−2^	3.52 × 10^−1^	1.01 × 10^−4^	0.4718
RC1	1.27 × 10^−4^	4.12 × 10^−2^	1.33 × 10^−2^	7.68 × 10^−2^	2.16 × 10^−1^	2.38 × 10^−4^	0.3477
RC2	1.63 × 10^−4^	6.55 × 10^−2^	2.13 × 10^−2^	7.68 × 10^−2^	2.37 × 10^−1^	1.97 × 10^−4^	0.4010
WC1	3.57 × 10^−5^	6.76 × 10^−2^	1.02 × 10^−2^	7.68 × 10^−2^	2.66 × 10^−1^	2.12 × 10^−4^	0.4208
WC2	3.66 × 10^−5^	6.68 × 10^−2^	8.77 × 10^−3^	7.68 × 10^−2^	3.54 × 10^−1^	2.43 × 10^−4^	0.5066
WC3	4.12 × 10^−5^	4.97 × 10^−2^	2.01 × 10^−2^	7.68 × 10^−2^	2.86 × 10^−1^	1.85 × 10^−4^	0.4328

## Data Availability

The original contributions presented in the study are included in the article, further inquiries can be directed to the corresponding author.

## Supplementary Materials

The following supporting information can be downloaded at https://www.mdpi.com/article/10.3390/foods13050742/s1, Figure S1: Activity of radioactive elements in black teas from India before and after the brewing process; Figure S2. Activity of radioactive elements in black teas from different parts of the world before and after the brewing process; Figure S3. Activity of radioactive elements in red teas from China before and after the brewing process; Figure S4. Activity of radioactive elements in oolong teas from China before and after the brewing process; Figure S5. Activity of radioactive elements in white teas from China before and after the brewing process; Figure S6. Elements content in black teas from India; Figure S7. Elements content in black teas from different parts of the world; Figure S8. Elements content in red teas from China; Figure S9. Elements content in oolong teas from China; Figure S10. Elements content in white teas from China.
