# Supplementary material for: Do You Know What You Drink? Comparative Research on the Contents of Radioisotopes and Heavy Metals in Different Types of Tea from Various Parts of the World
Source: Foods. 2024 Feb 28;13(5):742. doi: 10.3390/foods13050742 (PMC10931209; doi:10.3390/foods13050742)
Supplement: Supplementary file 1 [file foods-13-00742-s001.zip › foods-2878311-supplementary.pdf]

Supporting materials

Do You Know What You Drink? Comparative Research on the Contents of Radioisotopes and Heavy Metals in Different Types of Tea from Various Parts of the World

Elżbieta Grządka [1,*], Anna Bastrzyk [2], Jolanta Orzeł [1], Agata Oszczak-Nowińska [3], Bartłomiej Fliszkiewicz [3], Mateusz Siemieniuk [4], Krzysztof Sobczyński [4], Olgierd Spławski [4], Katarzyna Gołębiowska [1], Oskar Ronda [4] and Bartłomiej Michał Cieślik [4]

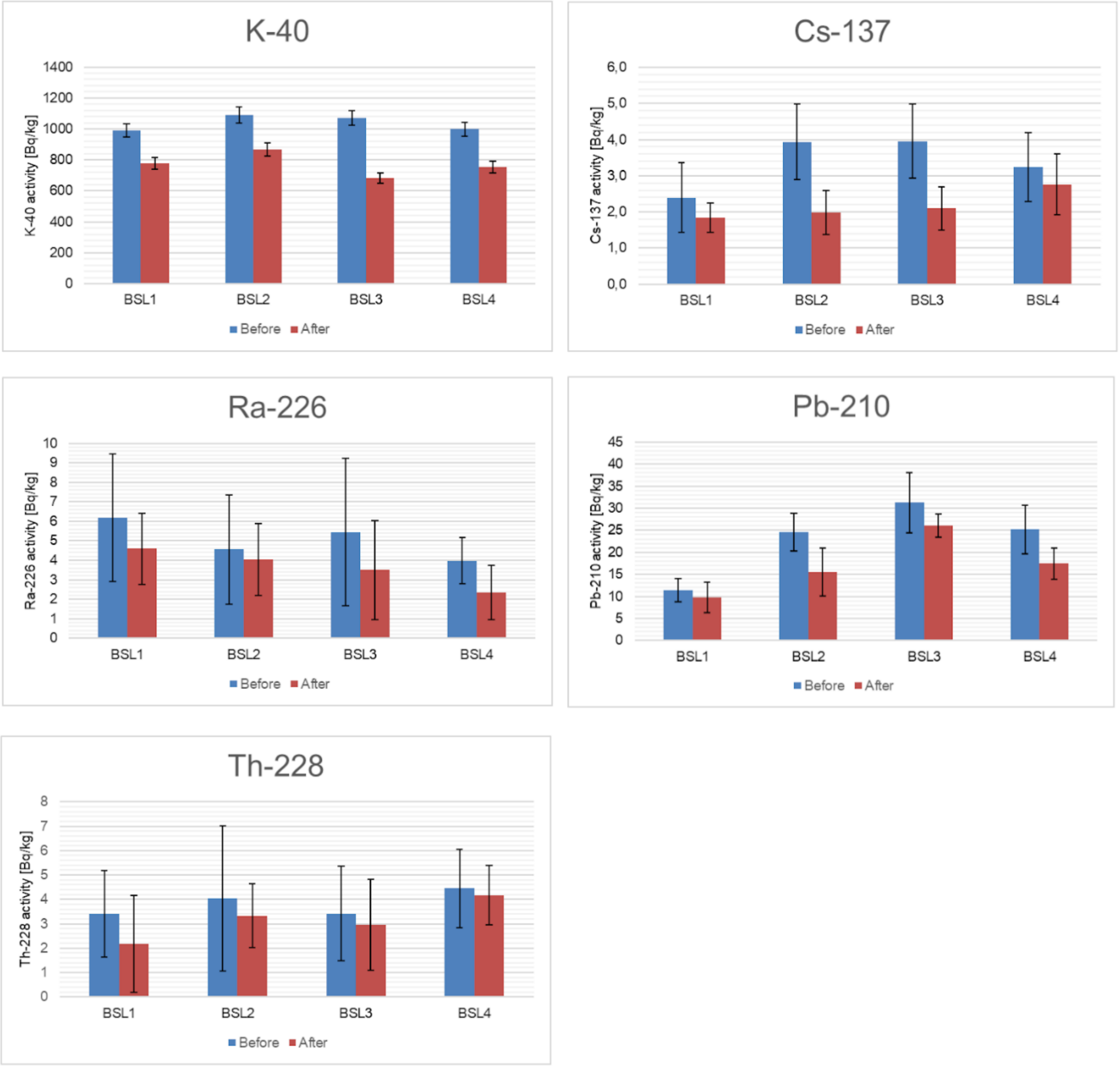

Figure S1. Activity of radioactive elements in black teas from India before and after the brewing process.

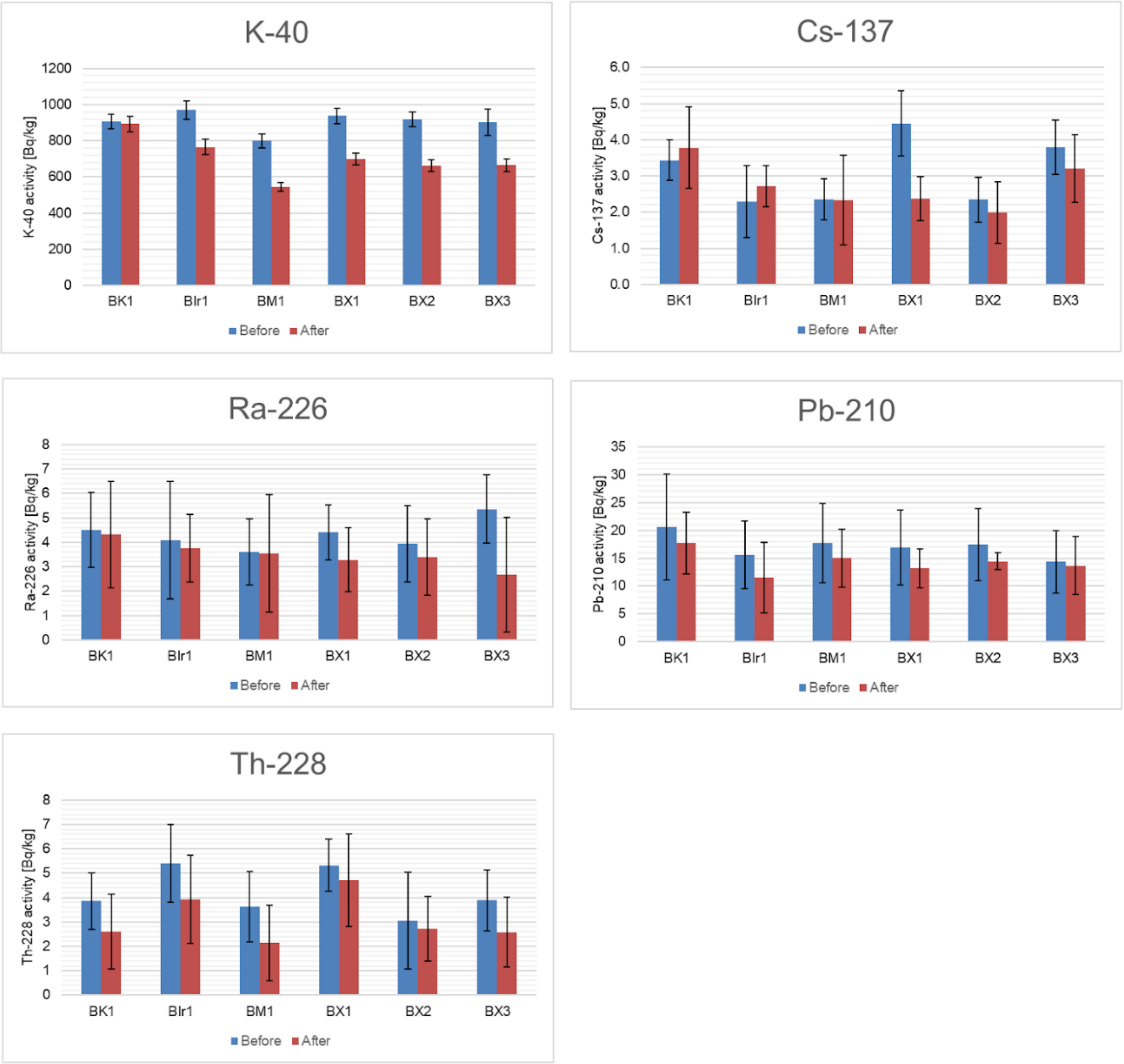

Figure S2. Activity of radioactive elements in black teas from different parts of the world before and after the brewing process.

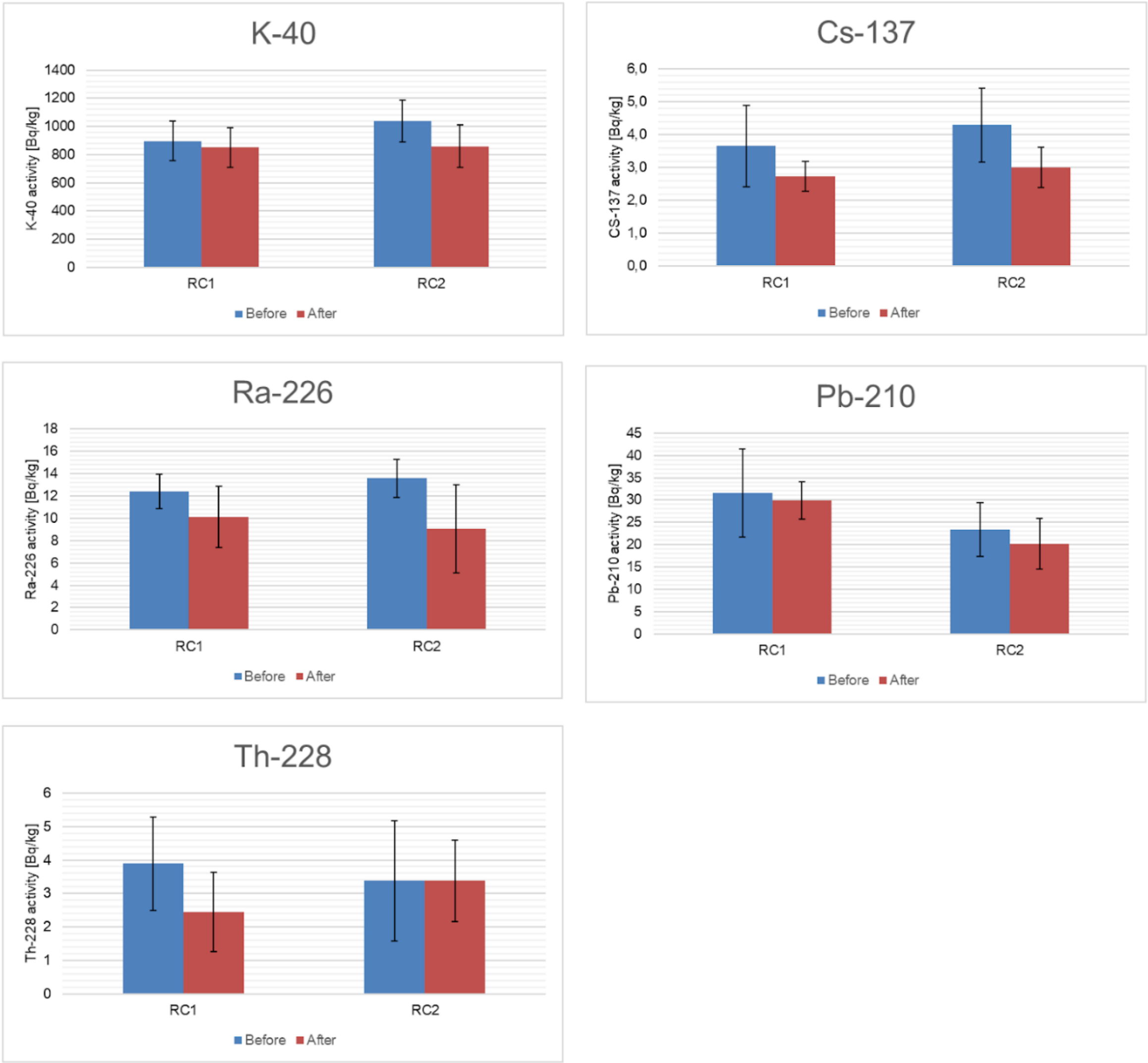

Figure S3. Activity of radioactive elements in red teas from China before and after the brewing process.

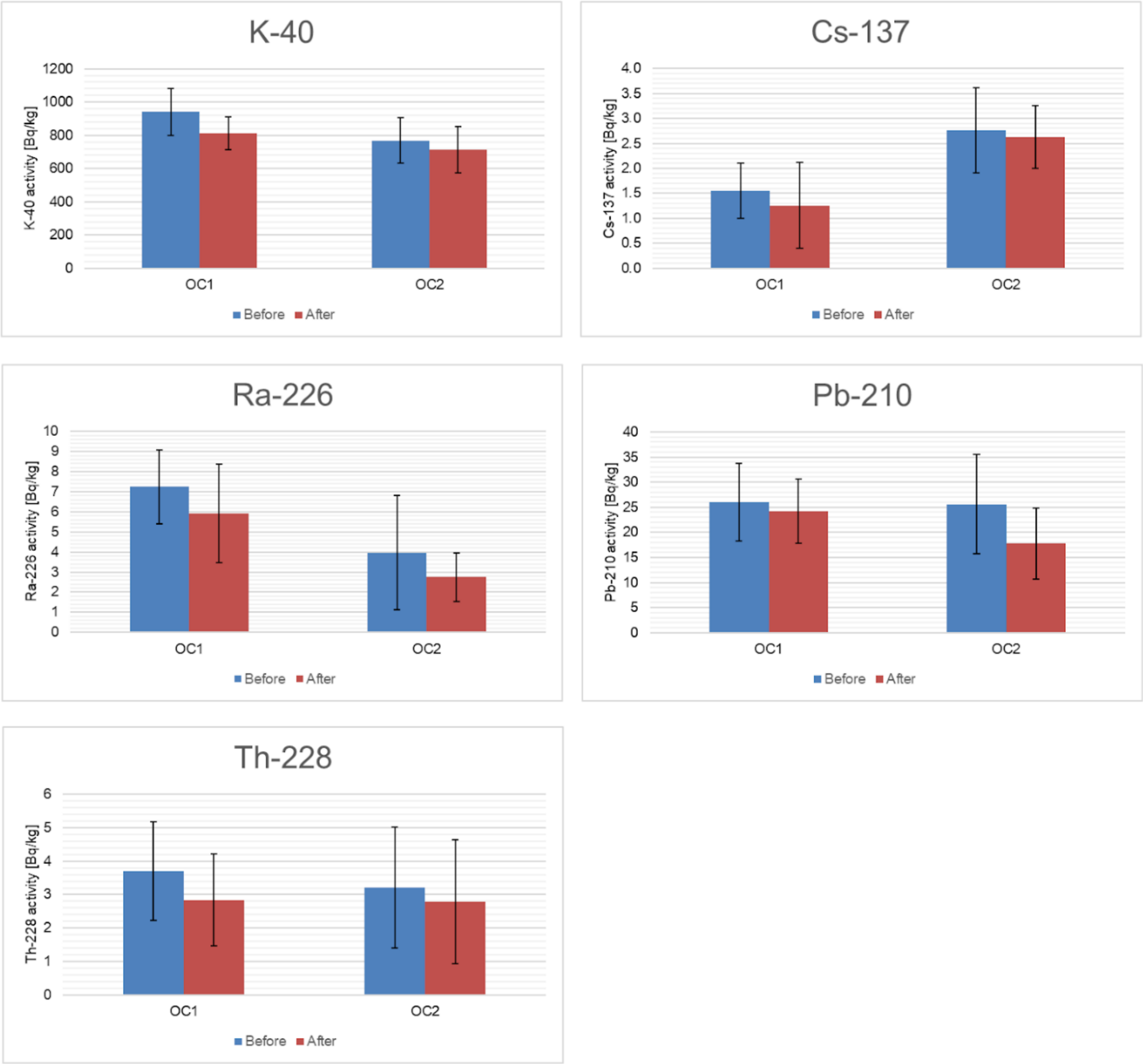

Figure S4. Activity of radioactive elements in oolong teas from China before and after the brewing process.

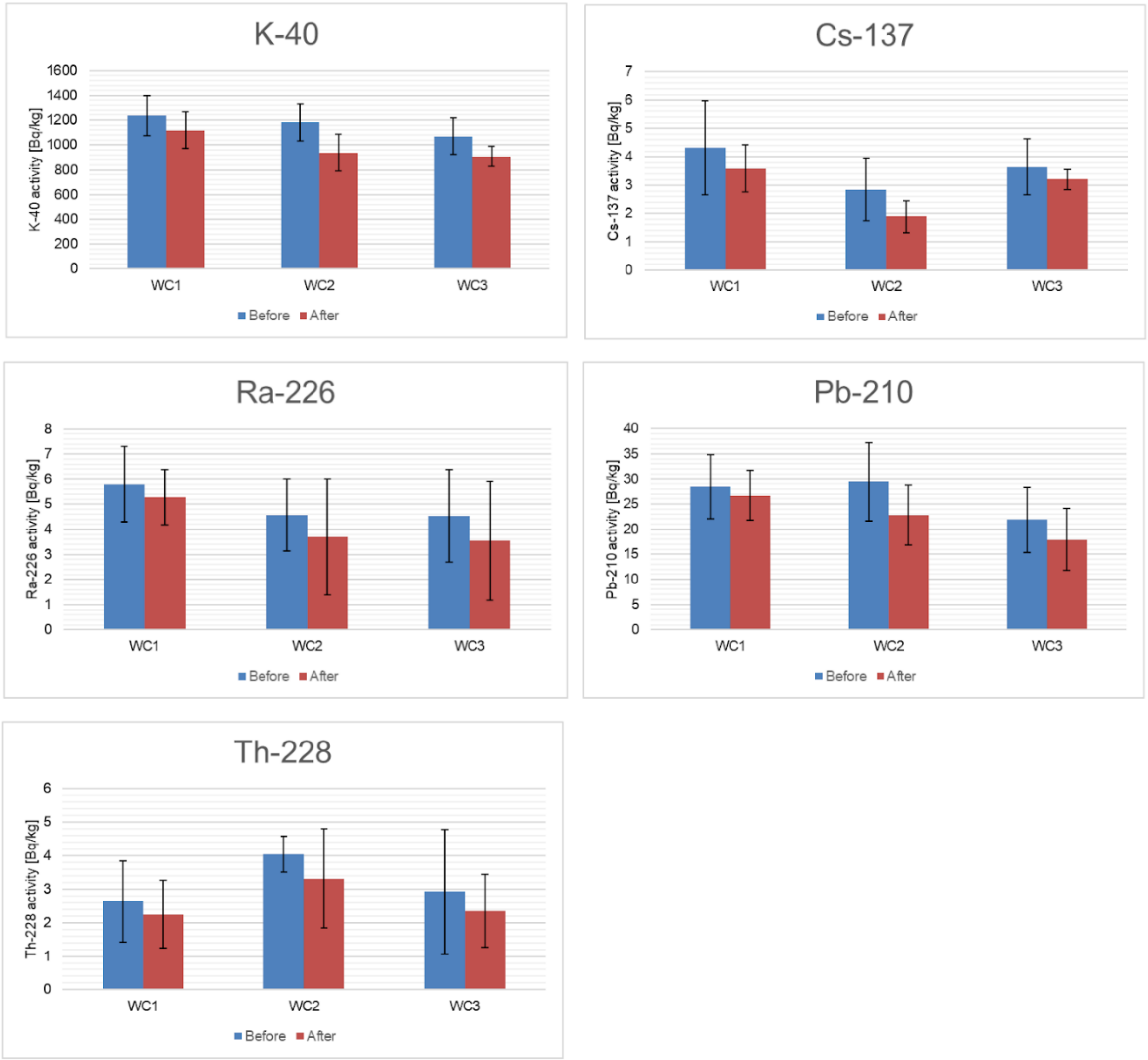

Figure S5. Activity of radioactive elements in white teas from China before and after the brewing process.

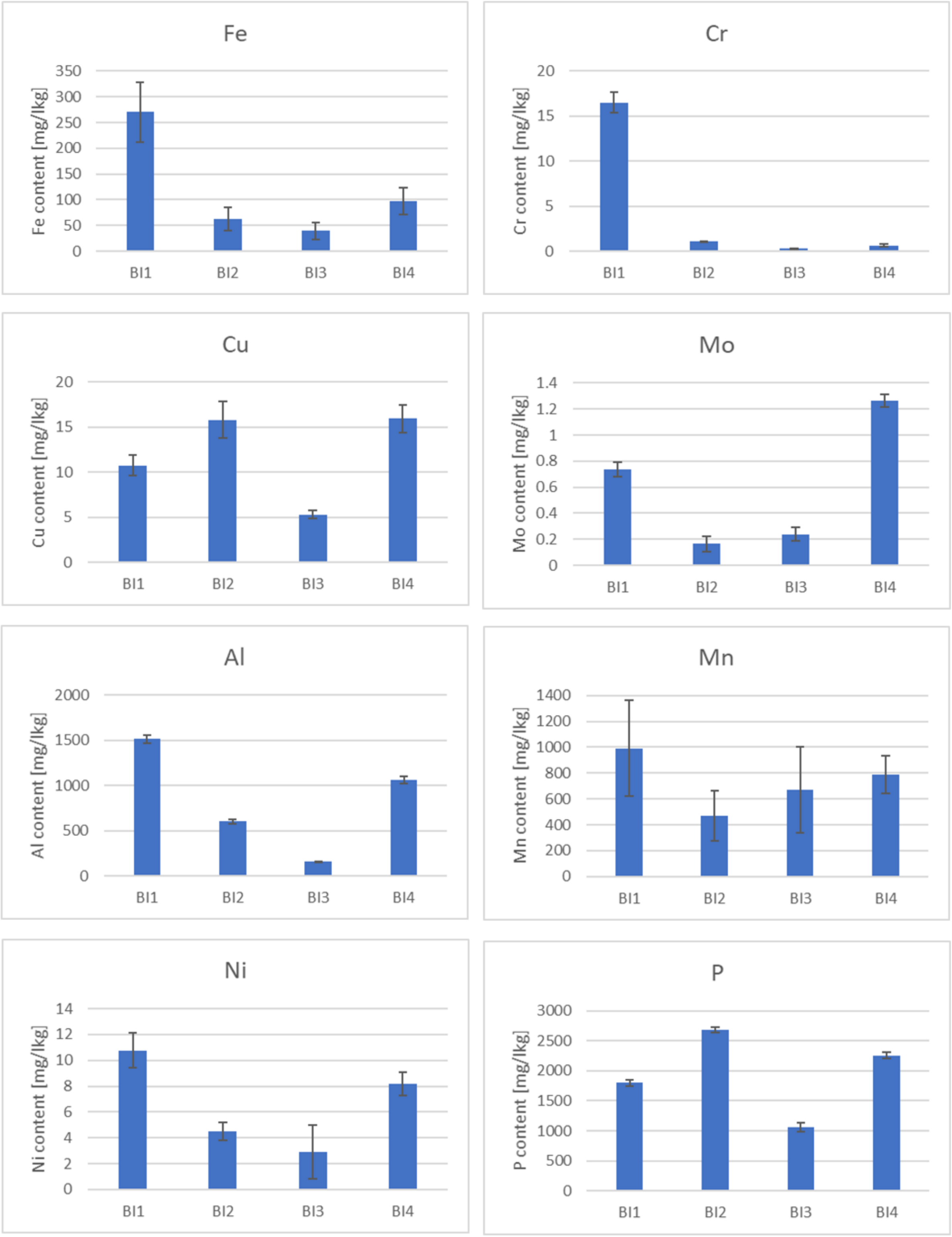

Figure S6. Elements content in black teas from India.

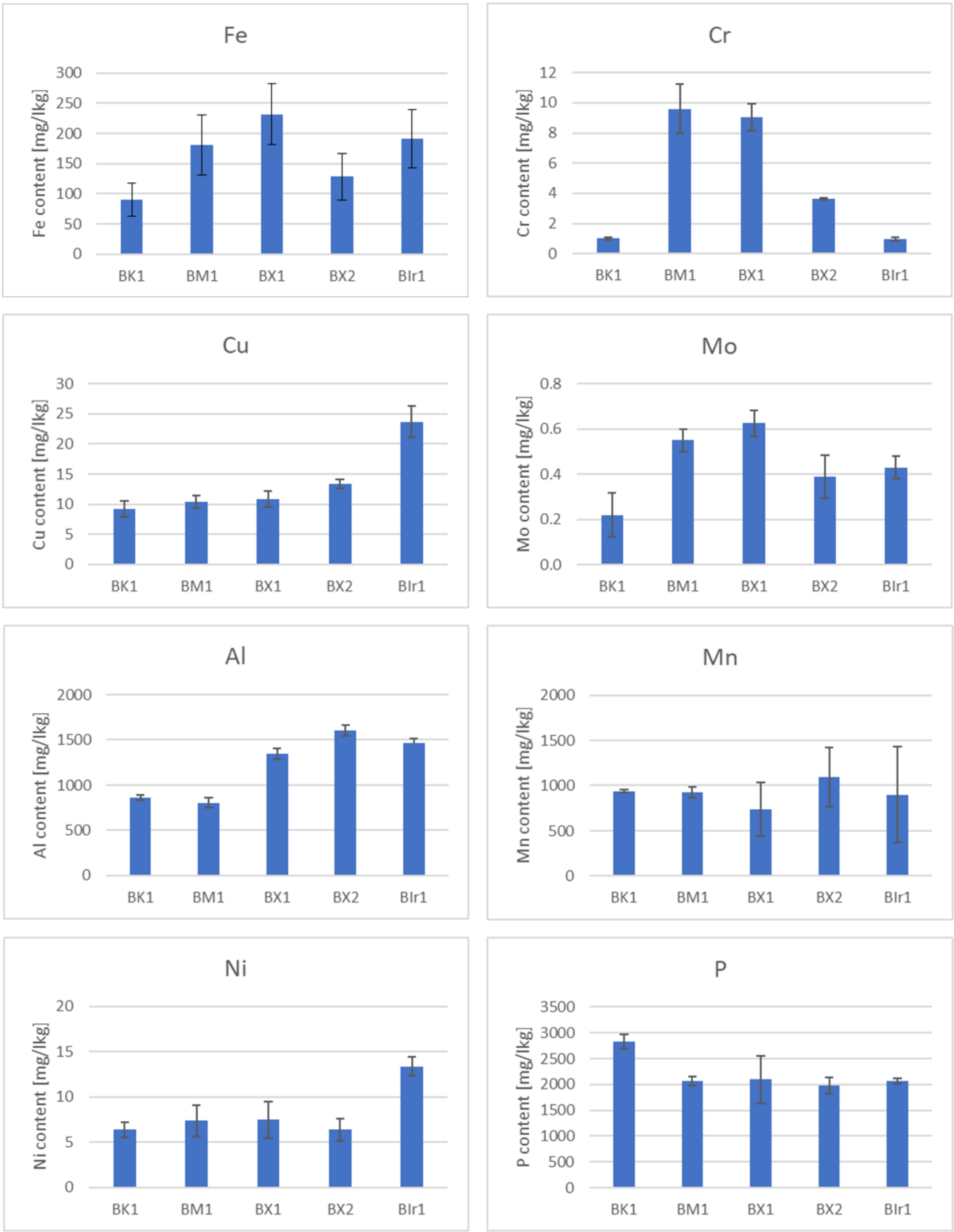

Figure S7. Elements content in black teas from different parts of the world.

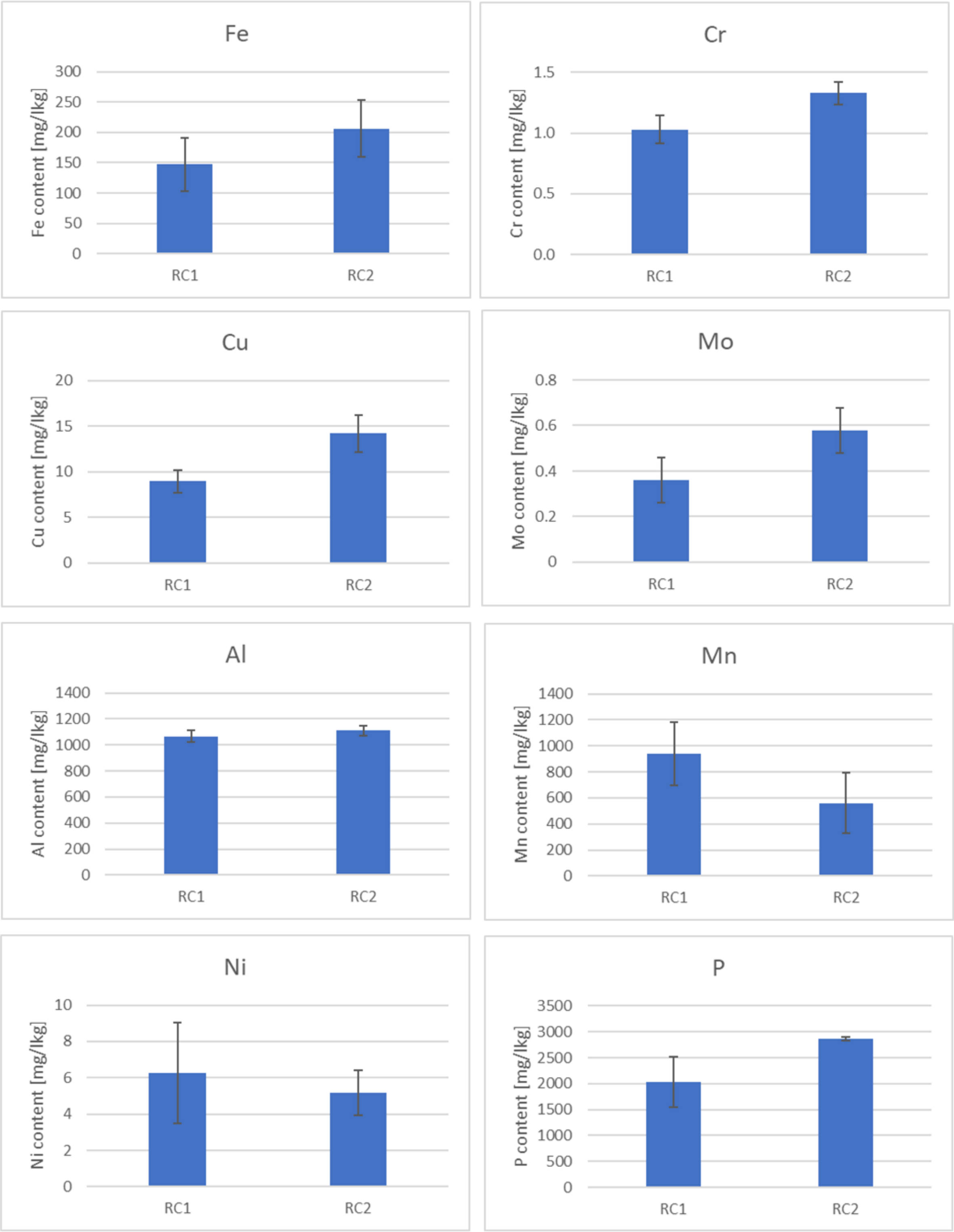

Figure S8. Elements content in red teas from China.

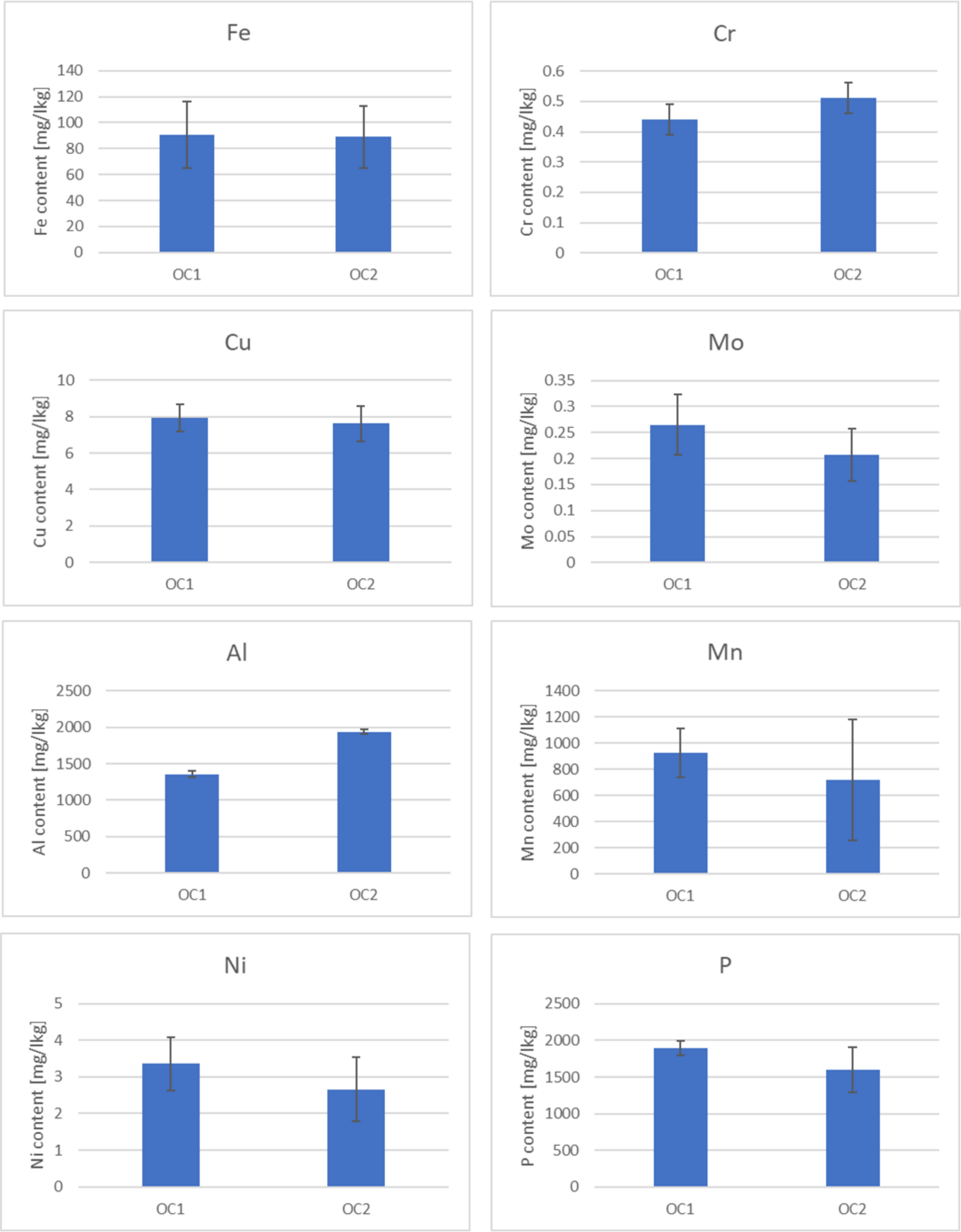

Figure S9. Elements content in oolong teas from China.

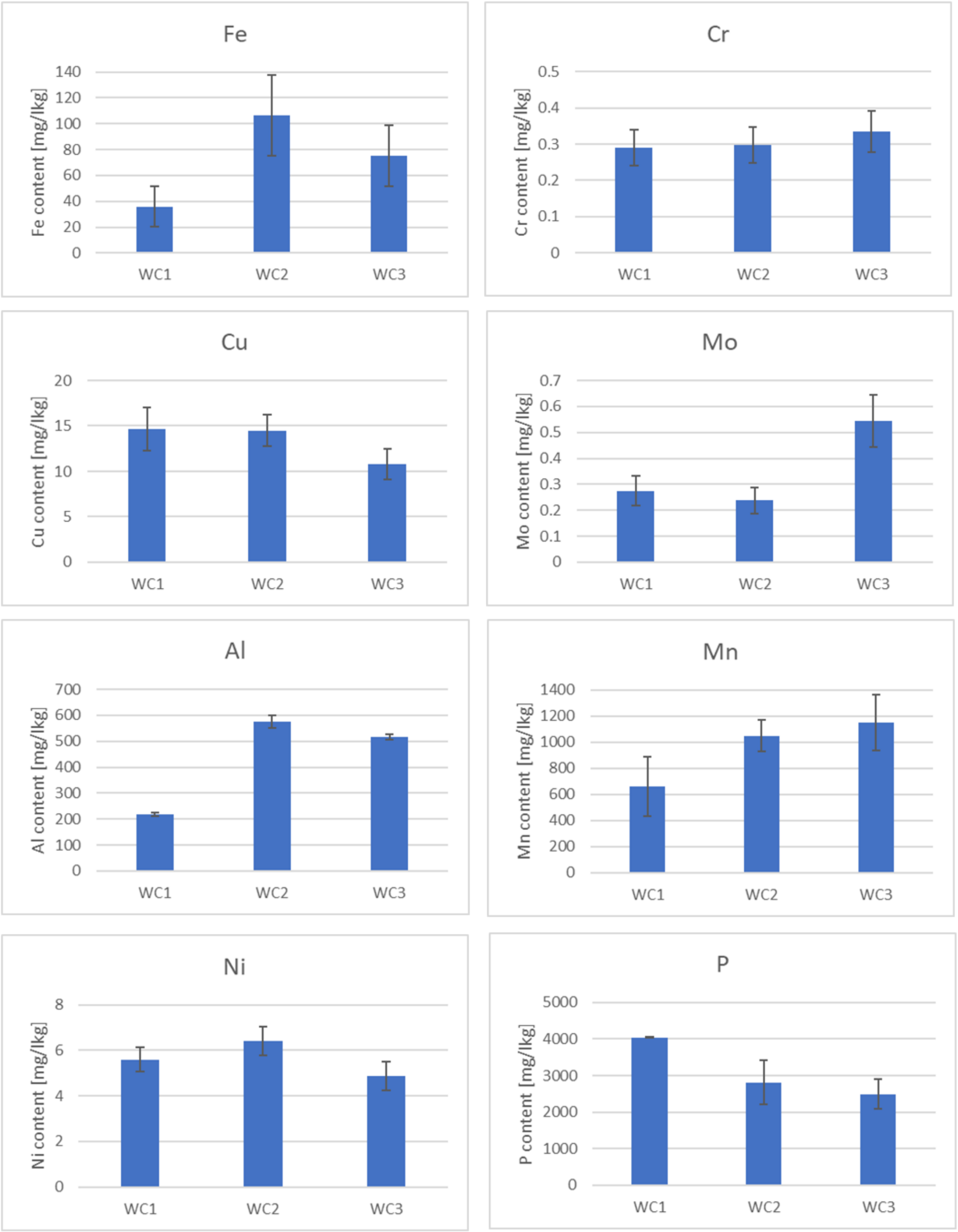

Figure S10. Elements content in white teas from China.
